# Circulating Tumor DNA as a Prognostic Biomarker for Recurrence in Patients With Locoregional Esophagogastric Cancers With a Pathologic Complete Response

**DOI:** 10.1200/PO.24.00288

**Published:** 2024-12-06

**Authors:** Eric Michael Lander, Vasily N. Aushev, Brandon M. Huffman, Diana Hanna, Punashi Dutta, Jenifer Ferguson, Shruti Sharma, Adham Jurdi, Minetta C. Liu, Cathy Eng, Samuel J. Klempner, Michael K. Gibson

**Affiliations:** ^1^ Minnesota Oncology Hematology PA, Minneapolis, MN; ^2^ Natera, Inc, Austin, TX; ^3^ Department of Medical Oncology, Dana-Farber Cancer Institute, Boston, MA; ^4^ USC Norris Cancer Center, Keck Medicine of USC, Los Angeles, CA; ^5^ Division of Hematology/Oncology, Vanderbilt-Ingram Cancer Center, Nashville, TN; ^6^ Department of Medicine, Division of Hematology-Oncology, Massachusetts General Hospital, Boston, MA; ^7^ Division of Hematology/Oncology, Department of Medicine, Vanderbilt University Medical Center, Nashville, TN

## Abstract

**PURPOSE:**

After neoadjuvant therapy (NAT) and surgery, up to one third and one half of patients with esophagogastric adenocarcinoma with a pathologic complete response (pCR; tumor regression grade 0 [TRG-0]) and near-pCR (TRG-1) will recur, respectively. Our study aims to evaluate postoperative circulating tumor DNA (ctDNA) as a predictor of recurrence in patients with pCR or near-pCR after curative-intent neoadjuvant chemotherapy or neoadjuvant chemoradiation and surgery.

**METHODS:**

We retrospectively identified patients from 11 institutions with stages I-IV esophagogastric cancers (EGCs) who completed NAT and had TRG-0/1 scores at the time of curative-intent surgery. Postoperative plasma samples were collected for ctDNA analysis within a 16-week molecular residual disease (MRD) window after definitive surgery, and during surveillance from January 7, 2020, to November 9, 2023, at the provider's discretion. ctDNA was assessed using a clinically validated, personalized, tumor-informed ctDNA assay (Signatera, Natera, Inc). The primary outcome was recurrence-free survival (RFS).

**RESULTS:**

We obtained 309 blood samples from 42 patients with esophagogastric adenocarcinoma with a pCR after neoadjuvant treatment over a median follow-up time of 28.5 months (range, 0.2-81.7). Detectable ctDNA in the 16-week MRD window (N = 23) correlated with higher rates of recurrence (67%; 2/3) compared with undetectable ctDNA (15%; 3/20). Detectable ctDNA within the MRD window was associated with a significantly shorter RFS (hazard ratio [HR], 6.2; *P* = .049). Among 32 patients who had ctDNA analyzed in the surveillance setting, the recurrence rate was 100% (5/5) in the ctDNA-positive cohort compared with 7.4% (2/27) in ctDNA-negative patients and was associated with shorter RFS (HR, 37.6; *P* < .001).

**CONCLUSION:**

Within the subgroup of patients with EGC and favorable pathologic responses (TRG 0-1) after NAT, the presence of postoperative ctDNA identified patients with elevated recurrence risk.

## INTRODUCTION

Esophagogastric cancer (EGC) remains the seventh leading cause of cancer-related mortality worldwide.^1^ Of the 60% of patients diagnosed with potentially curative locoregional disease in the United States, only 25%-30% survive 5 years from the time of diagnosis.^2^ The current management of locoregional EGC consists of perioperative neoadjuvant chemotherapy (NAC) or neoadjuvant chemoradiation (NCRT), followed by surgical resection and adjuvant chemotherapy for those who received NAC before surgery.

**CONTEXT**

**Key Objective**
Can postoperative circulating tumor DNA (ctDNA) status predict recurrence-free survival (RFS) in patients with esophagogastric cancer with a pathologic complete response (pCR) or near-pCR?
**Knowledge Generated**
Detectable ctDNA within the molecular residual disease and surveillance windows was associated with a significantly shorter RFS (hazard ratio [HR], 6.2; *P* = .049 and HR, 37.6; *P* < .001).
**Relevance**
ctDNA is prognostic for recurrence even in patients who achieved a pCR or near-pCR. With prospective validation in a larger cohort, ctDNA testing may become a useful surrogate modality along with other clinicopathologic factors to help identify patients who would benefit from adjuvant treatment.

After preoperative NCRT and surgery, approximately 23%-40% of patients who undergo resection will have no pathologic residual disease at the tumor bed or surrounding lymph nodes (ypT0, ypN0), classified as a pathologic complete response (pCR).^3,4^ Although achieving a pCR has been associated with improved overall survival (OS) and recurrence-free survival (RFS) compared with non-pCR, 17%-25% of patients with pCR experience recurrence within 2 years after surgery.^5-9^ Patients with esophageal adenocarcinoma who do not achieve pCR are eligible to receive adjuvant immunotherapy. The CheckMate 577 study demonstrated improved RFS with 1 year of adjuvant nivolumab for patients without a pCR; however, patients with a pCR were excluded from this trial.^10^ Thus, tailored surveillance strategies are needed to identify patients who, despite achieving pCR, remain at high recurrence risk.

Circulating tumor DNA (ctDNA) is a minimally invasive, blood-based biomarker that detects molecular residual disease (MRD) with high sensitivity and specificity in the absence of, or preceding, radiographic evidence of disease.^11-13^ Using a personalized, tumor-informed ctDNA assay for patients with locoregional EGC, Huffman et al^14^ found that ctDNA detection anytime post-operatively was highly prognostic of poor outcomes and disease recurrence. Their investigation, however, did not specifically evaluate the prognostic capabilities of ctDNA in patients with a favorable pCR result. Theoretically, ctDNA status may identify patients with a pCR at the highest risk of recurrence, potentially enabling early intervention leading to improved long-term outcomes.

In this study, we sought to specifically evaluate the performance of a tumor-informed ctDNA assay among patients with a pCR or near-pCR after NAC/NCRT using the cohort of patients gathered by Huffman and colleagues. We hypothesized that even within this favorable risk group, postoperative detectable ctDNA would be a strong predictor of RFS.

## METHODS

### Study Population

This retrospective analysis of real-world data included commercial ctDNA testing in patients (N = 212) with stages I-IV EGC from over 70 institutions. Demographic and clinicopathologic data regarding this patient cohort have been previously published.^14^ For this present investigation, 42 patients (19.8%, 42/212) from 11 institutions with tumor regression grade 0 (TRG-0)/pCR or TRG-1/near-pCR at the time of curative-intent surgery were included. All institutions used the American Joint Committee of Cancer and College of American Pathologists tumor regression grading system.^15^

Tumor tissue was obtained from biopsy or resected tumor for ctDNA assay design and development. Plasma samples were collected postoperatively (n = 309) during routine clinical care at the discretion of the ordering physician and analyzed within two separate windows: (1) an MRD window (within 16 weeks after surgery before initiation of adjuvant therapy) and (2) postoperative surveillance (>16 weeks after surgery excluding any on-treatment time points) during routine clinical follow-up from January 2020 until November 2023. This time interval for the MRD window was selected because of its clinical relevance for adjuvant immunotherapy initiation as used in Checkmate 577.^10,16^ ctDNA time points that were not followed by radiologic imaging were not used for the survival analysis. Clinicopathologic information was recorded from physician progress notes, laboratory results, and imaging reports. This study was granted institutional review board exempt with waiver status (institutional review board 221638) at each institution and was conducted per the Declaration of Helsinki.

### Personalized, Tumor-Informed, Polymerase Chain Reaction–Based Next-Generation Sequencing Assay for ctDNA Detection

MRD detection via ctDNA testing was performed using a clinically validated, personalized, tumor-informed, 16-plex polymerase chain reaction (PCR) next generation sequencing (NGS) assay (Signatera, Natera, Inc) as previously described.^13,14^ To design the personalized assay, whole-exome sequencing (WES) was performed on the formalin-fixed paraffin-embedded tissue block from patients' tumor and matched normal blood samples. Up to 16 high-ranked, patient-specific, somatic, single-nucleotide variants from WES were selected and designed for multiplex PCR (mPCR) testing and downstream NGS. A ctDNA-positive result was one in which the plasma sample harbored two or more tumor-specific gene variants above the calling confidence threshold. ctDNA concentration was reported as mean tumor molecules per mL of plasma.

### Statistical Analysis

The primary outcome measure was RFS, measured from the date of surgery to the first documented sign of radiologic recurrence, either locoregional or distant, and censored at the last follow-up or non–cancer-related death. Biopsies were not required to prove recurrence. Patient characteristics were summarized using descriptive statistics, and statistical significance was evaluated using Fisher's exact test for categorical variables. The Kaplan-Meier method was used for estimating the survival distributions. Hazard ratios (HRs) and associated 95% CIs and *P* values were calculated using Cox regression analysis. Log-rank test was used for comparing two survival distributions with *P* ≤ .05 being considered significant. Analysis was performed using the R statistical environment (v4.1.2).

## RESULTS

### Patient Characteristics

**FIG 1.**
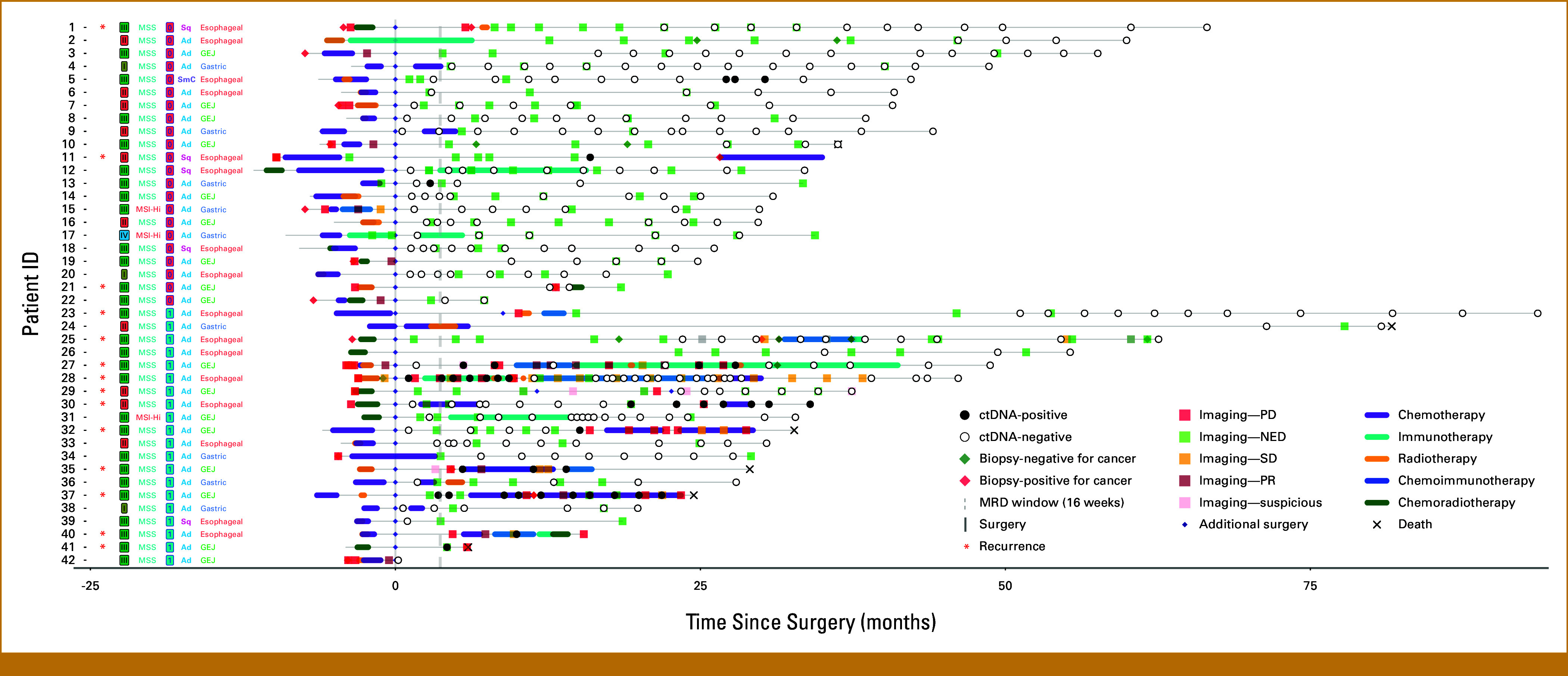
Swimmer's plot showing clinical outcomes, duration of systemic therapy, and longitudinal ctDNA analysis for 42 patients with stages I-IV esophagogastric cancers. Patients within each stage are ordered by decreasing clinical follow-up duration. Clinical stage, MSS/MSI status, TRG score, histology, and tumor type for every patient are listed by the patient ID on the *y*-axis. TRG-0: no viable cancer cells (complete response); TRG 1: single cell or rare small groups of cancer cells (near-complete response). Ad, adenocarcinoma; ctDNA, circulating tumor DNA; GEJ, gastroesophageal junction; Hi, high; ID, identification; MSI, microsatellite instability; MSI-H, microsatellite instability-high; MSS, microsatellite stable; NED, no evidence of disease; PD, progressive disease; PR, partial response; SD, stable disease; SmC, small cell carcinoma; Sq, squamous; TRG, tumor regression grade.

A total of 309 plasma samples were analyzed from 42 patients with esophageal (n = 16; 38.1%), gastroesophageal junction (GEJ; N = 17; 40.5%), and gastric (n = 9; 21.4%) cancers, with a median follow-up time of 28.5 months (range, 0.2-81.7). Of these, 85.7% (n = 36) were adenocarcinoma and 92.9% (n = 39) were microsatellite stable. Demographics and tumor characteristics are shown in Table 1. Figure 1 illustrates the clinical course of each patient in this cohort. At the time of surgical resection, 52% (22/42) of the patients had a TRG score of 0 and the remaining 48% (20/42) had TRG-1. After surgery, 23 patients had a ctDNA test available within the 16-week MRD window. Post-MRD time points were available for 41 patients, including on-treatment time points available for nine patients, and surveillance (post-MRD, not on-treatment) time points available for 32 patients.

**TABLE 1.** Demographic Table Highlighting Patient and Tumor Characteristics

Category	Patients (N = 42), No. (%)
Sex	
Female	9 (21.4)
Male	33 (78.6)
Cancer location	
Esophageal	16 (38.1)
GEJ	17 (40.5)
Gastric	9 (21.4)
TRG	
0	22 (52.4)
1	20 (47.6)
Stage at diagnosis	
I	3 (7.1)
II	10 (23.8)
III	28 (66.7)
IV	1 (2.4)
Histologic subtype	
Adenocarcinoma	36 (85.7)
Small cell	1 (2.4)
Squamous	5 (11.9)
Histologic grade	
G1	1 (2.4)
G2	7 (11.9)
G3	12 (28.6)
NA	24 (57.1)
Signet ring cells	
No	13 (31.0)
Yes	4 (9.5)
NA	25 (59.5)
HER2 status	
Negative	24 (57.1)
Positive	7 (16.7)
NA	11 (26.2)
PD-L1 CPS	
0	4 (9.5)
≤1	1 (2.4)
>1	11 (26.2)
NA	26 (61.9)
MSS/MSI status	
MSI-H	3 (7.1)
MSS	39 (92.9)
Neoadjuvant treatment	
Chemoradiotherapy	28 (66.6)
Chemotherapy	12 (28.6)
Chemoimmunotherapy	2 (4.8)

Abbreviations: CPS, combined positive score; GEJ, gastroesophageal junction; HER2, human epidermal growth factor receptor 2; MSI, microsatellite instability; MSI-H, microsatellite instability-high; MSS, microsatellite stable; NA, not available; TRG, tumor regression grade.

### Postoperative ctDNA Detection and Risk of Disease Recurrence

**FIG 2.**
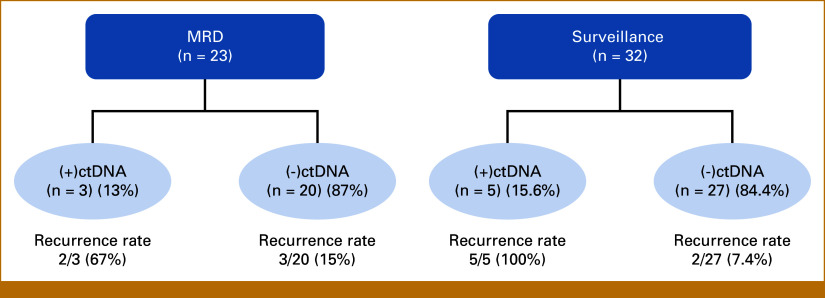
ctDNA status and recurrence rates for patients with ctDNA analyzed either in the 16-week MRD window or post-MRD surveillance window over a median follow-up of 28.5 months. ctDNA, circulating tumor DNA; MRD, molecular residual disease.

For patients analyzed in the postoperative 16-week MRD window (n = 23), 13% (3/23) tested positive for ctDNA, of whom 67% (2/3) experienced clinical recurrence. By contrast, of the remaining 87% (20/23) who had undetectable ctDNA, three (15%) relapsed (Fig 2). Upon evaluating post-MRD ctDNA status, 15.6% (5/32) of patients experienced a positive ctDNA test during surveillance. ctDNA positivity in the surveillance window was a strong predictor of recurrence: all five ctDNA-positive patients experienced clinical recurrence, while in 27 ctDNA-negative patients, only two (7.4%) relapse events were observed (mediastinal and locoregional lymph node recurrences).

Of the 20 patients who tested ctDNA-negative in the MRD window, 19 had subsequent ctDNA testing during disease surveillance. Of these 19 patients who were initially ctDNA-negative in the MRD window, three patients converted to ctDNA-positive in the post-MRD surveillance setting (patients 27, 30, and 32; Fig 1). Thus, 15.7% of patients who were initially ctDNA-negative later tested ctDNA-positive when evaluated during disease surveillance >16 weeks after surgery. Each of these three patients experienced disease recurrence, while none of the 16 patients with subsequent negative testing experienced disease recurrence.

**FIG 3.**
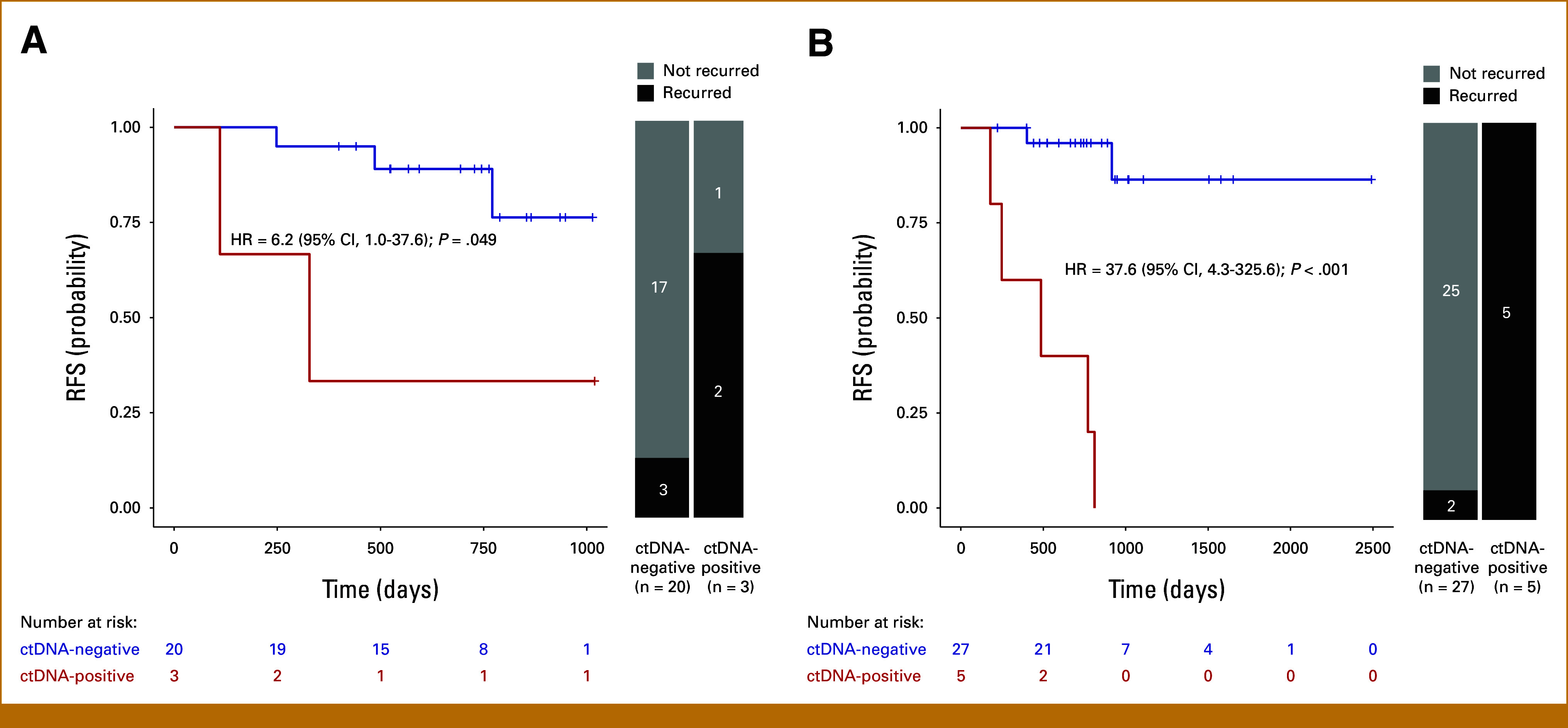
Association of ctDNA status with the probability of RFS. (A) Kaplan-Meier estimates for RFS stratified by ctDNA-negative and ctDNA-positive status for patients within the 16-week MRD window (N = 23). (B) Kaplan-Meier estimates for RFS stratified by ctDNA-negative and ctDNA-positive status for patients who had ctDNA analyzed at surveillance postoperatively (N = 32) over a median follow-up of 28.5 months. HRs and 95% CIs were calculated using the Cox proportional hazard model. *P* values were calculated using the two-sided log-rank test. ctDNA, circulating tumor DNA; HR, hazard ratio; MRD, molecular residual disease; RFS, recurrence-free survival.

Within the 16-week MRD window (n = 23), ctDNA positivity was associated with a significantly shorter RFS compared with undetectable ctDNA (HR, 6.2 [95% CI, 1.0 to 37.6]; *P* = .049; median RFS 10.7 months *v* not reached; Fig 3A). Similarly, in the postoperative surveillance setting, ctDNA-positive patients experienced a significantly worsened RFS compared with their ctDNA-negative counterparts (HR, 37.6 [95% CI, 4.3 to 325.6]; *P* < .001; median RFS 15.9 months *v* not reached; Fig 3B). Of the seven patients who had positive ctDNA before disease relapse, the median lead time from positive ctDNA testing to radiographic recurrence was 78 days.

### Association of ctDNA Status With Recurrence

Among the 22 patients with a pCR/TRG-0 (patient 1-22; Fig 1), 13 had ctDNA testing performed during the MRD window, of whom one tested positive (patient 13) at the limit of detection; however, that patient subsequently became ctDNA-negative and remained relapse-free during the follow-up period. None of the 12 patients who were ctDNA-negative at the MRD window relapsed. Of the 22 pCR/TRG-0 patients, all had ctDNA tested in the post-MRD surveillance window and 20/22 (90.9%) were ctDNA-negative and had no subsequent relapses. Of the two who tested ctDNA-positive, one patient (11) subsequently experienced a clinical relapse, while the other one (patient 5) had no confirmed relapse by the end of follow-up.

Among the subcohort of 20 patients who achieved a near-pCR/TRG-1 (patients 23-42; Fig 1), 11 had ctDNA testing within the MRD window; one patient (patient 42) was excluded from the MRD analysis because of lack of clinical follow-up after the ctDNA time point. Of these 10 patients, 20.0% tested ctDNA-positive (2/10) and both eventually developed radiographic recurrence. Upon evaluating post-MRD ctDNA status, ctDNA time points in the surveillance window were available for 12 patients, of whom 33.3% (4/12) tested ctDNA-positive. Notably, all these ctDNA-positive patients experienced recurrence (100%, 4/4), in contrast to only 12.5% (1/8) of patients with undetectable ctDNA (patient 25). Kaplan-Meier estimates of RFS were not performed for the pCR/TRG-0 and near-pCR/TRG-1 subgroups, given the limited sample size.

## DISCUSSION

We evaluated the real-world performance of a personalized, tumor-informed ctDNA assay in detecting MRD for patients with EGC who experienced the most favorable outcomes after neoadjuvant therapy (NAT) and surgery (pCR/TRG-0 or near-pCR/TRG-1). Even in the setting of minimal to no pathologic residual disease, ctDNA monitoring enabled MRD detection in some patients and was significantly prognostic of disease recurrence whether in the MRD window (HR, 6.2; *P* = .049) or during subsequent surveillance >16 weeks after surgery (HR, 37.6; *P* < .001). Our data suggest that ctDNA is prognostic for recurrence even in patients who achieved a pCR or near-pCR.

Emerging evidence shows the prognostic value of ctDNA in the postoperative setting for patients with EGC.^14,17-22^ However, to our knowledge, this is the first study to investigate the importance of longitudinal ctDNA monitoring in patients with a pCR. In 2024, this remains a relevant clinical issue since the decision to treat patients with adjuvant therapy is currently gated by whether patients achieve a pCR.^23^ The CheckMate577 study, which established the benefit of adjuvant immunotherapy for patients with esophageal and GEJ adenocarcinoma who received NCRT, only included patients without a pCR.^10^ Therefore, patients with a pCR are not currently eligible for adjuvant therapy, although 17%-25% of these patients will experience metastatic disease recurrence within 2 years after surgery.^5-9^ KEYNOTE-585, a randomized, double-blind, phase III trial (ClinicalTrials.gov identifier: NCT03221426), calls into question the ability of pCR in the primary tumor to serve as a surrogate for RFS/OS. Our data suggest that ctDNA may complement and improve further stratification along with pCR if used in this setting. We observed a median lead time of 78 days from positive ctDNA testing to radiographic recurrence. When validated in larger cohorts, the clinical incorporation of ctDNA testing may further refine risk stratification after NAT and surgery with potential implications for the direction of adjuvant therapy. ctDNA status was evaluated to detect MRD within 16 weeks after surgery, during which critical clinical decisions are made regarding identifying potential candidates for adjuvant therapy.^16,24^ In this cohort, ctDNA positivity was detected in 13% of patients (3/23), of whom 67% (2/3) experienced disease recurrence. The remaining patient (patient 12 in Fig 1) had an insufficient clinical follow-up. On the contrary, only 15% of patients (3/20) with negative ctDNA testing in the MRD window experienced disease recurrence. All three of these patients who experienced recurrence developed detectable ctDNA >16 weeks after surgery in the surveillance setting before their recurrence, reflecting an additional 15% of patients who developed positive ctDNA testing with longitudinal monitoring.

Recently, results from ESOPEC trial comparing perioperative FLOT with CROSS regimen demonstrated improved OS with FLOT (median OS 66 months *v* 37 months; HR, 0.70 [0.53-0.92]; *P* = .012) and higher pCR with FLOT (16.8% *v* 10%).^25^ Although the results of this trial may result in more patients with EGC being treated with FLOT, neoadjuvant CRT remains a viable option for patients with localized EGC, as evidenced by the lack of superiority of either regimen in a subgroup analysis of ESOPEC in patients with clinical T1-2 or N0 stage. Furthermore, the results of this trial do not account the results of CheckMate577 trial where adjuvant Nivolumab after CROSS regimen in patients with incomplete pCR resulted in doubling the median DFS compared with placebo. Given all of the above, CROSS regimen remains a viable option for patients with earlier-stage disease or who cannot tolerate FLOT, and ctDNA can be used in that setting to assess MRD and for recurrence monitoring, even among patients with pCR.

Given the real-world nature of this study, several limitations should be noted. There may be inherent selection bias regarding which patients the providers selected for testing. The timing of ctDNA testing and imaging evaluations for recurrence were heterogeneous across the cohort. The sample size was relatively small, limiting the ability to assess survival outcomes for TRG-0 and TRG-1 scores independently. Although many patients had a shorter follow-up that limited evaluation for recurrence in some cases, the median follow-up of 28.5 months still represents a clinically meaningful time frame since most patients with a pCR who do recur will do so within 2 years. We observed a small number of patients with negative ctDNA who still experienced disease recurrence. This may be explained by a subset of tumors that could have minimal ctDNA shedding into the bloodstream. Since providers may have modulated their radiographic imaging schedules on the basis of ctDNA results, the lead time from ctDNA positivity to traditional imaging recurrence may be inaccurate. One small retrospective study identified a median lead time of 1 year with the tumor-informed ctDNA assay; however, the study did not specifically evaluate the lead time in patients with a pCR.^21^

In summary, our study shows that postoperative ctDNA is prognostic of recurrence in a real-world population of patients with EGC with a pCR/near-pCR (TRG-0/TRG-1) at the time of surgery after NAT. If prospectively validated in a larger cohort, ctDNA testing may become a useful surrogate modality along with other clinicopathologic factors to help identify patients with EGC who would benefit from adjuvant treatment.

## Data Availability

A data sharing statement provided by the authors is available with this article at DOI https://doi.org/10.1200/PO.24.00288.
